# The intraoperative SEEG and ^18^FDG-PET tailored temporal lobe resection (iSP-TR) for the tissue-spearing surgical treatment of drug-resistant mesial temporal lobe epilepsy: how we do it

**DOI:** 10.1007/s00701-025-06491-x

**Published:** 2025-03-19

**Authors:** Francesca Battista, Riccardo Carrai, Antonello Grippo, Alessandro Della Puppa

**Affiliations:** 1 Department of Neurosurgery, Careggi University Hospital, Florence, Italy; 2 Neurophysiopathology Unit, Neuro-Muscular-Skeletal Department, Careggi Hospital, University of Florence, Florence, Italy; 3 Department of Neurosurgery, Department of Neuroscience, Drug Area and Child Health (NEUROFARBA), University of Florence, Careggi University Hospital, PsychologyFlorence, Italy

**Keywords:** Meningiomas, iSEEG, Epilepsy, Epilepsy surgery, FDG-PET, Temporal lobe epilepsy

## Abstract

**Background:**

For drug-resistant mesial temporal lobe epilepsy (MTLE), surgery is recommended. There is no evidence of a seizure outcome advantage of temporal lobectomy over other strategies despite the high risks of this surgical procedure.

**Methods:**

We describe a Temporal Resection guided by intraoperative stereo-electroencephalography (iSEEG) and the ^18^FluoroDeoxyGlucose PET (^18^FDG PET) (iSP-TR) to treat lesional MTLE. We avoided functional cortical areas using navigated Transcranial Magnetic Stimulation (nTMS).

**Conclusion:**

iSP-TR allows for a spare brain parenchyma compared to temporal lobectomy without compromising seizure outcomes.

**Supplementary Information:**

The online version contains supplementary material available at 10.1007/s00701-025-06491-x.

## Relevant surgical anatomy

Mesial temporal lobe epilepsies (MTLE) are the most common forms of focal epilepsy[2]. The temporal lobe's higher frequency of epileptogenic foci may be due to its structure[3]. Unlike other cerebral lobes, the temporal lobe consists of a lateral portion of the neocortex, which often contains essential functional cortical areas, such as those responsible for language or hearing, and a mesial portion composed of different types of allocortex and mesocortex. The hippocampus and the dentate gyrus are composed of the archicortex (3 layers); the entorhinal and piriform cortices consist of the paleocortex (3–5 layers); the subiculum and part of the entorhinal cortex are made up of mesocortex (4–5 layers); the parahippocampal gyrus and the temporal pole are composed of the neocortex (6 layers). The distinct evolutionary age and unique structural characteristics of the mesial temporal lobe regions contribute to their higher susceptibility to the development of epileptogenic foci[3].

The temporal lobe has widespread connections to nearby and distant cortical and subcortical structures. There is a strong connection between the two hippocampi [4] and its connections with the insula, thalamus, caudate, and ipsilateral temporal-lateral cortical structures[10]. These findings have been made possible through studies on patients who underwent temporal lobectomy for drug-resistant epilepsy, revealing that such surgery leads to omo- and bilateral cortical thinning[1] and atrophy[4] in structures both adjacent to and distant from the surgical cavity. Furthermore, several studies using ^18^FluoroDeoxyGlucose-PET (^18^FDG-PET) imaging have also observed the onset of hypometabolism in cortical areas adjacent to the surgical cavity[5, 6]. This reinforces the concept that while temporal lobectomy can improve seizure outcomes, it may also disrupt the functional neuronal network through mechanisms of degeneration and remote atrophy[7], and this may be due to cognitive deficit. Therefore, it is crucial to develop surgical strategies that minimize the extent of brain tissue removal in temporal resections for epilepsy, aiming to preserve the functional network in which the temporal lobe serves as a central hub as much as possible.

## Description of the technique

**Fig. 1:**
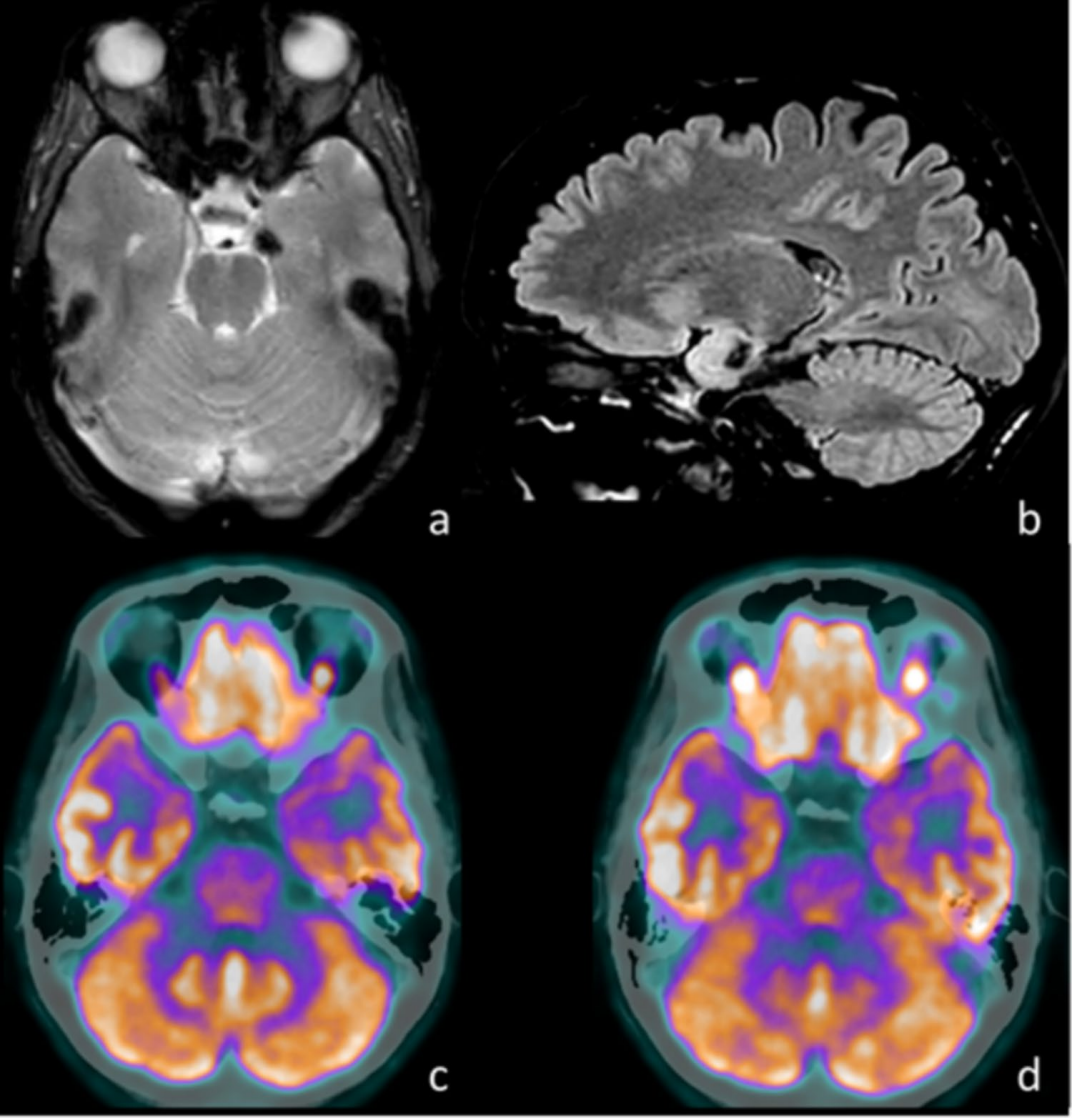
Illustrative case 1, drug-resistant MTLE due to left uncus cavernoma: **a** MRI, T2-weighted axial sequence, showing signal hypointensity of the left uncus; **b** MRI, FLAIR sagittal sequence, highlighting signal hypointensity of the left uncus; **c** ^18^FDG-PET, demonstrating marked hypometabolism in the left uncus and temporal pole compared to the contralateral side; **d** ^18^FDG-PET, demonstrating hypometabolism in the head and body of the left hippocampus compared to the contralateral side. MRI – Magnetic Resonance Imaging; ^18^FDG-PET: ^18^FluoroDeoxyGlucose Positron Emission Tomography; FLAIR – Fluid-Attenuated Inversion Recovery

**Fig. 2:**
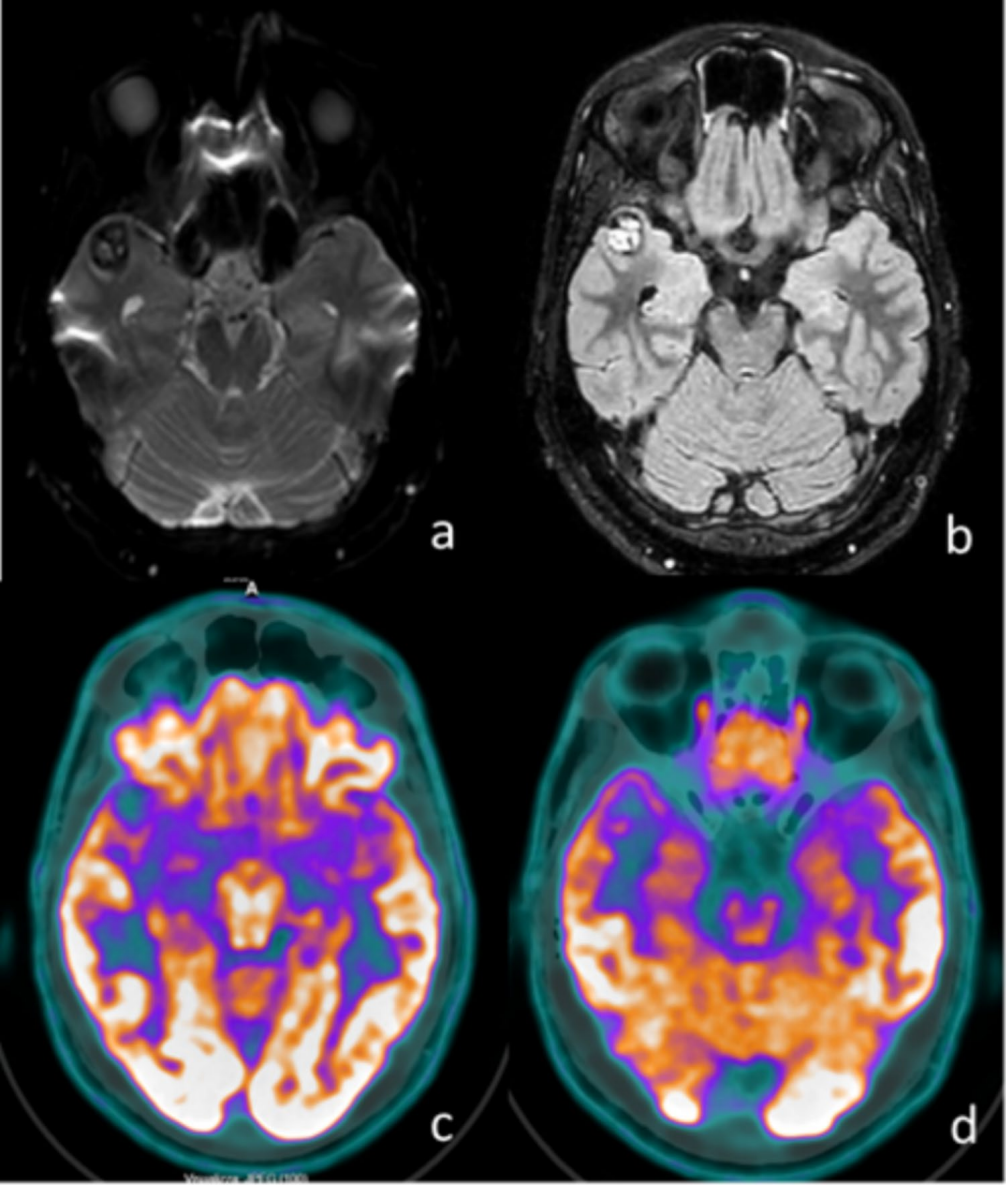
Illustrative case 2, drug-resistant MTLE due to right temporal pole cavernoma: **a** MRI, DWI-weighted axial sequence, showing signal hypointensity of the right temporal pole; **b** MRI, FLAIR sagittal sequence, highlighting signal hyperintensity of the right temporal pole; **c** ^18^FDG-PET, demonstrating marked hypometabolism in the right temporal pole compared to the contralateral side; **d** ^18^FDG-PET, demonstrating marked cortical thinness and hypometabolism in right temporal pole compared to the contralateral side. MRI – Magnetic Resonance Imaging; DWI: Diffusion-weighted image; ^18^FDG-PET: ^18^FluoroDeoxyGlucose Positron Emission Tomography; FLAIR – Fluid-Attenuated Inversion Recovery

We describe the temporal resection guided by intraoperative stereo-electroencephalography (iSEEG) and ^18^FDG-PET hypometabolism (iSP-TR) for the treatment of drug-resistant MTLE due to temporal-mesial epileptogenic lesions (Figs. 1 and 2 report two example cases underwent iSP-TR). Magnetic resonance imaging (MRI) and ^18^FDG-PET imaging are coregistered and uploaded to neuronavigator.

Intraoperative monitoring of motor-evoked potentials (MEP) is conducted for motor function. Functional cortical areas (i.e., language) are identified using navigated Transcranial Magnetic Stimulation (nTMS). The patient underwent preoperative neuropsychological testing. The anesthetic protocol we use involves total intravenous anesthesia (TIVA), with the suspension of propofol and overlapping administration of dexmedetomidine after dural opening, allowing for intraoperative electrocorticography (iECoG) and iSEEG signal assessment. The patient is supine, with the omolateral shoulder elevated by support and the head fixed using the Mayfield system, rotated 40° to the contralateral side. The temporal lobe is exposed using a pterional approach. Upon dural opening, the exposed functional language areas at nTMS are identified to ensure their preservation during resection.

To provide example images, we describe the intervention of illustrative case 1. As a first step, we placed an iECoG strip at multiple points on the lateral neocortex and selected the site with the most prominent interictal activity to monitor its evolution during resection.

**Fig. 3:**
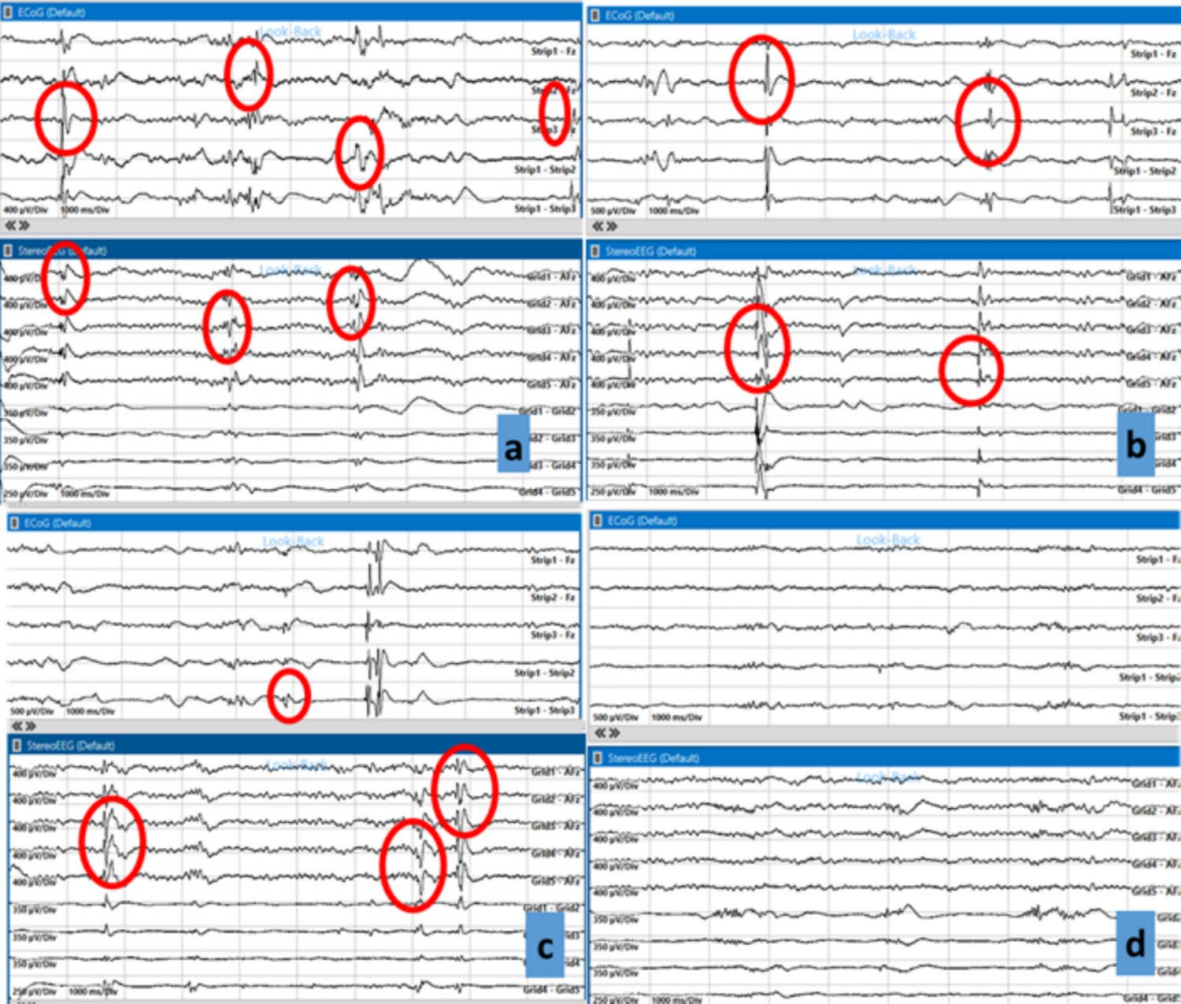
Illustrative case 1, images of iECoG and iSEEG during the stages of temporal resection: **a** Initial screen: frequent interictal abnormalities are evident both in surface iECoG and in iSEEG at the level of the uncus cavernoma; **b** Interictal abnormalities persist after lesionectomy in both iECoG and iSEEG; **c** iSEEG in the head of the hippocampus shows evident interictal abnormalities, while surface iECoG fails to detect them, as these are temporal- mesial structures; **d** Final screen: interictal abnormalities have disappeared. iECoG: intraoperative electrocorticography; iSEEG: intraoperative stereo-electroencephalography

Before initiating the resection, we recorded iSEEG using a stereo-electroencephalographic electrode, which helps explore mesial temporal or intrasulcal areas that are difficult to assess with iECoG strips. An iSEEG electrode was first positioned at the uncus level, where the cavernoma was located, to record activity before resection, revealing prominent interictal discharges (Fig. 3a). The cavernoma and the associated hemosiderin ring were then resected (lesionectomy). After repeating iSEEG and iECoG, we observed that the interictal activity remained unchanged from the pre-lesionectomy recordings. Using neuronavigation, we noted that the ^18^FDG-PET hypometabolism was primarily concentrated in the uncus and temporal pole (Fig. 2c). Consequently, the iSEEG electrode was placed at the temporal pole, where we re-recorded prominent interictal activity. A temporal polectomy of approximately 2 cm was performed, leading to a slight reduction in pathological activity upon subsequent iSEEG and iECoG monitoring (Fig. 3b). Following the ^18^FDG-PET findings, we observed additional hypometabolism in the head and body of the hippocampus (Fig. 2d). iSEEG recordings at the hippocampal level revealed prominent interictal activity. Notably, at this stage of surgery, the iECoG signal recorded from the lateral neocortex was still present but reduced compared to the initial recordings, and it was unable to detect the hippocampal anomalies captured by iSEEG (Fig. 3c). Therefore, a subpial resection of the hippocampal head and body was performed, with repeated iSEEG monitoring showing a progressive reduction of interictal activity, eventually nearing complete disappearance. A further decrease in pathological interictal activity was also noted on iECoG. However, due to the persistence of high-frequency interictal anomalies on iECoG, an additional iSEEG recording was performed at the posterior limit of the polectomy on the neocortical side, revealing a high frequency of interictal discharges. After further assessment with nTMS, we expanded the lateral neocortical resection by approximately 0.5 cm, resulting in an almost complete disappearance of anomalies on both iSEEG and iECoG. Final iSEEG and iECoG assessments confirmed the stable disappearance of interictal anomalies (Fig. 3d). The patient did not experience any neurological deficits in the postoperative period. Postoperatively, a slight reduction in memory functions was observed, with significant recovery already noted at the one-month follow-up. The patient achieved complete seizure freedom at the one-month follow-up (Engel IA).

## Indications

The iSP-TR is indicated for patients with drug-resistant MTLE and associated lesions, such as mesial temporal cavernomas or hippocampal sclerosis, and the iSEEG can spare from preoperative classic SEEG surgery, which requires general anesthesia, allowing for less invasiveness for the patient.

Lesional MTLE cases are often treated with an extensive temporal lobectomy, involving a lateral resection extending 4.5 cm for the non-dominant hemisphere and 6.5 cm for the dominant hemisphere[8]. However, this approach, which relies on anatomical landmarks without intraoperative electroencephalographic data, exposes patients to a high risk of functional and cognitive deficits.

The temporal lobe also serves as a hub for connections with nearby and distant structures. The preservation of as much brain tissue as possible—both for functional purposes and maintaining the physiological connectome—is crucial, and iSP-TR represents a valid alternative to anatomical temporal lobectomy. This approach prioritizes sparing functional and non-functional healthy tissue without compromising seizure outcome success.

## Limitations

The main limitation is using the iSP-TR for MRI-negative MTLE, in which preoperative SEEG planning is still recommended, as intraoperative iSEEG alone cannot accurately define the seizure onset zone in these cases.

## How to avoid complication

Potential complications of iSP-TR include functional (language or motor) and cognitive deficits. To minimize the risk of language deficits, nTMS is used to preserve functional cortical areas. MEP monitoring is performed throughout the resection to prevent motor deficits. Like selective amygdalo-hippocampectomy (sAH)[9], iSP-TR aims to be less invasive, reducing the rate of postoperative cognitive deficits, even temporary ones, compared to temporal lobectomy.

## Specific information for the patient

The patient must be informed about the risk of cognitive deficits (such as attention and memory impairments) and neurological deficits (aphasia or hemiparesis). Additionally, the patient should be aware of the risk of seizure recurrence, as well as the need to continue antiseizure drugs for at least one-year post-surgery and, in the worst-case scenario, for life.

## Key points summary

1. Preserving tissue (both functional and non-functional) during temporal lobectomy improves the patient's postoperative cognitive performance.
2. The electrode for iSEEG allows recording of the electrical activity of every cortical area, including those that ECoG strips cannot reach (e.g., the depths of cortical sulci, the hippocampus).
3. The placement of the iSEEG electrode is performed either through visual control of the structures or guided by the neuronavigator.
4. Having at least 3–4 electrode contacts for iSEEG along the cortical thickness is essential to ensure a reliable signal recording.
5. In selected cases of lesional focal epilepsy, iSEEG can sometimes replace classical SEEG.
6. In cases of recording and resection of mesial temporal regions with iSEEG, keeping a strip on the lateral portion of the temporal lobe to monitor the evolution of the ECoG trace of the cortex during resection is useful.
7. ^18^FDG-PET hypometabolic areas in patients with drug-resistant MTLE often correspond to cortical areas with prominent interictal activity.
8. nTMS is a valuable tool for preserving functional cortical areas in asleep surgery, particularly in patients with drug-resistant epilepsy.
9. iSP-TR is a tissue-sparing, patient-tailored temporal resection technique that provides optimal seizure outcomes while preserving cognitive and functional abilities for lesional MTLE
10. iSP-TR is insufficient for patients with MTLE and negative MRI, requiring a thorough preoperative SEEG study.

## Supplementary Information

Below is the link to the electronic supplementary material.

ESM 1 (MP4 458 MB)

## Data Availability

No datasets were generated or analysed during the current study.
